# Seabird diversity hotspot linked to ocean productivity in the Canary Current Large Marine Ecosystem

**DOI:** 10.1098/rsbl.2016.0024

**Published:** 2016-08

**Authors:** W. James Grecian, Matthew J. Witt, Martin J. Attrill, Stuart Bearhop, Peter H. Becker, Carsten Egevang, Robert W. Furness, Brendan J. Godley, Jacob González-Solís, David Grémillet, Matthias Kopp, Amélie Lescroël, Jason Matthiopoulos, Samantha C. Patrick, Hans-Ulrich Peter, Richard A. Phillips, Iain J. Stenhouse, Stephen C. Votier

**Affiliations:** 1 Institute of Biodiversity, Animal Health and Comparative Medicine, University of Glasgow, Glasgow G12 8QQ, UK; 2 Environment and Sustainability Institute, University of Exeter, Penryn, Cornwall TR10 9EZ, UK; 3 Centre for Ecology and Conservation, University of Exeter, Penryn, Cornwall TR10 9EZ, UK; 4 Marine Institute, Plymouth University, Plymouth, Devon PL4 8AA, UK; 5 Institut für Vogelforschung ‘Vogelwarte Helgoland’, An der Vogelwarte 21, Wilhelmshaven 26386, Germany; 6 Greenland Institute of Natural Resources, Kvioq 2, 3900 Nuuk, Greenland; 7 Institut de Recerca de la Biodiversitat (IRBio) and Departament de Biologia Animal, Universitat de Barcelona, Av. Diagonal 643, Barcelona 08028, Spain; 8 CEFE UMR 5175, CNRS—Université de Montpellier—Université Paul-Valéry Montpellier—EPHE, 1919 route de Mende, 34293 Cedex 05, Montpellier, France; 9 DST/NRF Centre of Excellence, Percy FitzPatrick Institute, University of Cape Town, Rondebosch 7701, South Africa; 10 Institute of Ecology, Friedrich-Schiller University, 07743 Jena, Germany; 11 School of Environmental Sciences, University of Liverpool, Liverpool L69 3GP, UK; 12 British Antarctic Survey, Natural Environment Research Council, Cambridge CB3 0ET, UK; 13 Biodiversity Research Institute, 276 Canco Road, Portland, ME 04103, USA

**Keywords:** biologging, human impacts, marine protected areas, migration, upwelling, marine conservation

## Abstract

Upwelling regions are highly productive habitats targeted by wide-ranging marine predators and industrial fisheries. In this study, we track the migratory movements of eight seabird species from across the Atlantic; quantify overlap with the Canary Current Large Marine Ecosystem (CCLME) and determine the habitat characteristics that drive this association. Our results indicate the CCLME is a biodiversity hotspot for migratory seabirds; all tracked species and more than 70% of individuals used this upwelling region. Relative species richness peaked in areas where sea surface temperature averaged between 15 and 20°C, and correlated positively with chlorophyll *a*, revealing the optimum conditions driving bottom-up trophic effects for seabirds. Marine vertebrates are not confined by international boundaries, making conservation challenging. However, by linking diversity to ocean productivity, our research reveals the significance of the CCLME for seabird populations from across the Atlantic, making it a priority for conservation action.

## 1. Introduction

Upwelling regions are globally important marine biodiversity hotspots. The mixing of nutrient-rich cool water with warm surface layers fuels primary production, driving bottom-up cascades that also support large communities of upper trophic-level consumers [1]. As a result, they are attractive foraging grounds targeted by a wide-range of marine animals throughout the annual cycle [2]. These characteristics make upwelling regions strong candidates for protection, but this is challenging as they often cross national boundaries, occur in international waters and protection may conflict with fisheries interests [3].

Marine environments are facing unprecedented levels of anthropogenic-driven pressure; including climate change, pollution and offshore development [4–6]. The foremost threat to upwelling regions is biodiversity loss through overfishing; upwellings cover less than 1% of the world's ocean by area but provide approximately 20% of global catch [7]. Commercial capture fisheries deplete stocks, remove top-predators through bycatch, and alter the trophic structure of ecosystems [8,9]. The Canary Current Large Marine Ecosystem (CCLME) now incorporates one of the most intensively fished areas on the Earth [8,10], yet also supports large populations of migratory marine vertebrates from breeding populations across the Atlantic [11–13].

Considering the increasing industrialization of fisheries [10], the pervasive threat from bycatch [14] and a paucity of quantitative information on habitat or space use, understanding marine vertebrate distributions in the CCLME and beyond is a key conservation goal [15]. In this study, we use miniaturized light loggers to reconstruct the non-breeding movements of eight migratory seabird species from disparate regions of the Atlantic that have been previously recorded in the CCLME [12]. Our aims are: (i) to map the distribution of these birds and identify areas of high diversity, (ii) to quantify the extent to which each species uses the CCLME, and (iii) to determine the oceanographic characteristics that drive this association. We use our findings to assess the importance of the CCLME as a biodiversity hotspot and discuss the potential conflict between fisheries and seabirds in this region.

## 2. Material and methods

We collated data on the non-breeding movements of eight seabird species; Cory's shearwaters (*Calonectris borealis*); Scopoli's shearwaters (*C. diomedea*); lesser black-backed gulls (*Larus fuscus*); northern gannets (*Morus bassanus*); great skuas (*Stercorarius skua*); south polar skuas (*S. maccormicki*); common terns (*Sterna hirundo*) and Sabine's gulls (*Xema sabini*). While these species have been recorded previously in the CCLME, the true importance of this region for specific populations is unknown. Between 2000 and 2011, 123 birds were tracked using miniaturized light loggers from 12 breeding colonies from the north (75° N) to the south (62° S) of the Atlantic (see the electronic supplementary material). To quantify the extent to which each species uses the CCLME, we calculated the proportion of time each individual spent in this region [16]. To identify areas of high species richness we constructed spatial density maps by binning location data into 200 km diameter tessellated hexagons spanning the Atlantic. We calculated relative richness by summing the number of species occurring in each hexagon during the non-breeding period.

To characterize the marine environment, we extracted winter seasonal climatology composites (December–March, 2002–2010) of sea surface temperature (SST, °C) and chlorophyll *a* concentration (CHL, mg m^−3^) from the MODIS instrument onboard the Aqua (EOS PM) satellite (http://oceancolor.gsfc.nasa.gov/) and calculated mean SST and CHL values for each hexagon. We also included a measure of null usage that incorporated both habitat availability and sampling effort, as this was not uniform across species or colonies [17] (see the electronic supplementary material). These data are available via Dryad [18].

We examined correlations between the observed patterns in relative richness and these covariates using generalized additive models fitted with the packages mgcv [19] and MuMIn [20] in R v. 3.1.0 [21]. We log_10_ transformed CHL prior to use. We included SST, CHL and null usage as covariates in the global model with thin plate regression splines fitted with a maximum of 10 knots; superfluous knots were penalized during model fitting. Variance inflation factors revealed no multicollinearity between covariates (VIF < 3). We also included the central *X* and *Y* coordinates of each hexagon as a spatial smooth term implemented with a soap film boundary [22]. The soap film specifies the extent of the predicted surface, preventing smoothing across boundary features such as the Iberian Peninsula. Variograms of model residuals revealed no spatial autocorrelation in final models. Model selection was based on Akaike's information criterion (AIC), with parameters excluded if their inclusion did not improve the model by more than 2 ΔAIC relative to the lowest AIC.

## 3. Results

**Figure 1.**
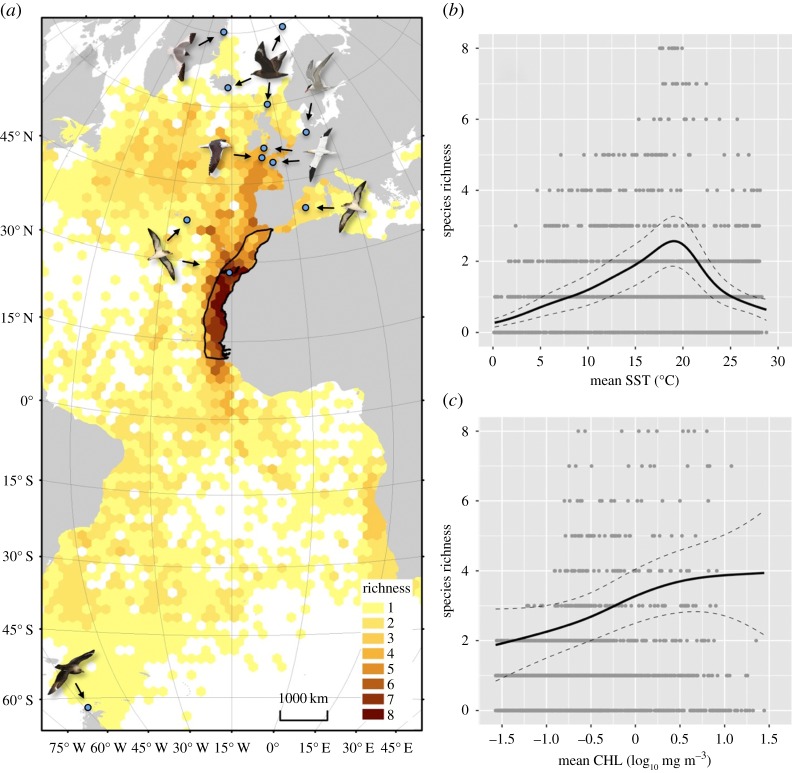
Links between (*a*) relative richness of eight seabird species tracked from pan-Atlantic colonies between 2000 and 2011; and (*b*) sea surface temperature (SST) and (*c*) chlorophyll *a* (CHL). Dark line in (*a*) represents the boundary of the Canary Current Large Marine Ecosystem; blue dots represent colonies of origin for tracked birds, indicated by arrows. Dark lines in (*b*) and (*c*) represent model-estimated response; dashed lines 95% confidence interval; light grey dots indicate the distribution of data. (Online version in colour.)

The eight species tracked from 12 colonies over 10 years were widely distributed across the Atlantic during the non-breeding period (figure 1). Highest relative richness was observed in the CCLME, with other hotspots in the Bay of Biscay, Mid-Atlantic Ridge, Brazilian coast and Benguela Current (figure 1). On average, 76.6 ± 28.1% of individuals from each species visited the CCLME, including all Scopoli's shearwaters, Sabine's gulls, south polar skuas and common terns, the majority of lesser black-backed gulls and northern gannets, but only 25% of great skuas (table 1). The proportion of time each species spent in the CCLME was highest for Scopoli's shearwaters (0.35 ± 0.28), northern gannets (0.26 ± 0.29) and common terns (0.24 ± 0.22). There was a high degree of variation both within and among species; individuals may use the CCLME for the entire non-breeding period, only as a staging area, or not at all (table 1).

**Table 1.** Summary statistics for tracking by seabird species. (Values represent mean ± s.d. For full methods and description of winter period, see the electronic supplementary material.)

species	*N*	% visiting CCLME	winter period (days)	no. locations	locations in CCLME	proportion in CCLME
lesser black-backed gull	7	71.4	208.4 ± 27.4	151.1 ± 20.8	21.0 ± 24.0	0.09 ± 0.10
northern gannet	34	58.8	93.6 ± 13.4	89.6 ± 15.4	25.7 ± 29.8	0.26 ± 0.29
great skua	16	25	92	91.9 ± 0.4	11.1 ± 21.8	0.12 ± 0.24
Cory's shearwater	19	57.9	133.9 ± 29.9	131.5 ± 27.4	13.8 ± 23.2	0.10 ± 0.18
Scopoli's shearwater	9	100	104.5 ± 40.8	102.3 ± 39.6	35.4 ± 36.9	0.35 ± 0.28
Sabine's gull	7	100	287.9 ± 12.7	228.6 ± 18.6	22.3 ± 3.1	0.08 ± 0.01
south polar skua	19	100	237.2 ± 35.1	176.3 ± 21.6	8.7 ± 14.3	0.04 ± 0.07
common tern	12	100	254.3 ± 67.0	181.3 ± 64.0	62.8 ± 51.8	0.24 ± 0.22

Relative richness correlated with SST and CHL; both terms were retained in the top-ranked model along with the soap film smooth term and measure of null usage (table 2). Model-estimates indicated relative richness was highest in areas with SST between 15 and 20°C, and there was a general positive correlation between relative richness and CHL (figure 1).

**Table 2.** Model selection testing correlations between relative richness and sea surface temperature (SST) and chlorophyll *a* (CHL). (The full model included a soap film smooth term (*XY*) and measure of habitat availability (null). Models shown are those within 6 ΔAIC of the best-supported model. Adj *R*^2^ of best-supported model = 0.60.)

rank	parameters	d.f.	AIC	ΔAIC
1	SST + CHL + *XY* + null	173	5573	0.00
2	SST + *XY* + null	175	5576	2.84
3	SST + CHL + *XY*	171	5578	4.47
4	SST + *XY*	173	5579	6.28

## 4. Discussion

Here, we demonstrate that the CCLME is an area of high relative species richness for non-breeding seabirds, and detail the environmental conditions that drive this association. More than 70% of individuals from eight species, representing a range of functional groups and originating from breeding colonies across the Atlantic, visited this upwelling region. Relative richness correlated with both SST and CHL. By tracking birds of known origin, our results illustrate the high connectivity between seabird breeding populations across the Atlantic and the CCLME, emphasizing the importance of this upwelling region as a non-breeding destination and migratory stopover site.

This study represents the most comprehensive collation of tracking data for the CCLME to date, but our measure of relative species richness is limited to those populations included in the study. While many other species also visit this region [12], modern developments in biologging are revealing a diversity of migration strategies [23] and highlighting other important areas across the Atlantic. Our measure of relative species richness represents the maximum across the study period and is likely to vary over the annual cycle in response to seasonal differences in environmental conditions. For example, Southern Hemisphere migrants following the austral summer overlap only briefly with Northern Hemisphere migrants in the CCLME (electronic supplementary material, table S1). While our study highlights the CCLME as a hotspot for migratory seabirds, further work is required to understand the significance of other areas across the Atlantic and beyond.

These findings provide evidence of the links between biodiversity and ocean productivity in an eastern boundary upwelling region. Relative richness was highest between 15 and 20°C, and correlated positively with CHL; corroborating previous work on the oceanographic drivers of marine predator diversity in the California Current [2]. This suggests that primary productivity in the CCLME has bottom-up effects that are highly relevant to apex predators. The mechanisms by which animals may target these regions are currently unknown, but frontal density in the CCLME is high and these visible indicators of productivity are known to aggregate marine predators such as seabirds [11,24].

The CCLME attracts some of the highest global fishing effort [8,10], yet there is a paucity of information on the interactions between seabirds and fisheries in this region [15]. Fisheries impact seabirds in three ways; either competing directly for fish, providing food in the form of discarded fish, or posing the threat of bycatch mortality [14,25,26]. More research into fine-scale, species-specific fisheries interactions in the CCLME is required, especially given recent evidence of direct take of seabirds in the region (K. Camphuysen 2013, personal communication); the substantial under-reporting of catch in this area by China's distant-water fleet [27]; and the prevalence of illegal, unreported and unregulated fisheries [28].

Integrating data across multiple species and years highlights the importance of the CCLME as a seabird biodiversity hotspot. Furthermore, environmental conditions such as SST and productivity may offer insights into how distributions could shift in response to global climate change. As marine vertebrates forage across dynamic pelagic systems and are not confined by international boundaries, effective conservation will require multilateral cooperation. Nevertheless, while site fidelity to persistent upwelling regions such as the CCLME could aid conservation, it is unlikely that both a large diversity of marine vertebrates and intense fisheries exploitation can be sustained in this region in the long term.

## Supplementary Material

Electronic Supplementary Materials

## Data accessibility

The species richness and environmental covariate data underlying this study are archived in the Dryad digital repository at http://dx.doi.org/10.5061/dryad.01sd0. Individual species data are archived by BirdLife International (www.seabirdtracking.org).
