# Supplementary material for: Seabird diversity hotspot linked to ocean productivity in the Canary Current Large Marine Ecosystem
Source: Biol Lett. 2016 Aug;12(8):20160024. doi: 10.1098/rsbl.2016.0024 (PMC5014014; doi:10.1098/rsbl.2016.0024)
Supplement: Electronic Supplementary Materials [file rsbl20160024supp1.docx]

**Supplementary methods**

In June 2008, we deployed 40 Mk 7 British Antarctic Survey (BAS) light geolocation sensors (GLS) on adult northern gannets (*Morus bassanus*) breeding on Ile Rouzic, Brittany, France (48° 54′ N, 3° 26′ W). In July 2009 we deployed 20 Mk 5 BAS GLS on adults breeding on Grassholm, Wales (51° 43′ N, 05° 28′ W). During 2010, 21 loggers (52.5%) were retrieved from Rouzic, 13 (65%) from Grassholm. In June 2009 we deployed 20 Mk 5 BAS GLS on adult lesser black-backed gulls (*Larus fuscus*) breeding on Gugh, Isles of Scilly, UK (49.891N, 6.330W), recovering 7 (35%) in 2010.

The non-breeding movements of 7 Sabine’s gulls (*Xema sabini*) tracked from a breeding colony on Sand Island (74° 73′ N, 20° 27′ W), Young Sound, Northeast Greenland between 2007 and 2008 were taken from Stenhouse, Egevang and Phillips [1]. Information on the non-breeding movements of 16 great skuas (*Stercorarius skua*) tracked by GLS from three breeding colonies in the northeast Atlantic; Bjørnøya in Svalbard, Norway (74° 29′ N, 18° 49′ E), Öræfi, Iceland (63° 52′ N, 16° 29′ W) and Foula in Shetland, Scotland (60° 08′ N, 2° 05′ W) between 1^st^ of November 2008 and 1st of February 2009 were taken from Magnusdottir *et al.* [2]. The movements of 19 adult South polar skuas (*Stecorarius maccormicki*) tracked between 2006 and 2008 from a breeding colony on King George Island in the Maritime Antarctic (62° 12′ S, 58° 58′ W) were taken from Kopp *et al.* [3]. The movements of 12 Common terns (*Sterna hirundo*) tracked from a breeding colony on Wilhelmshaven, German North Sea coast (53° 36’ N, 08° 06’ E) between 2010 and 2011 were taken from Becker *et al.* [4]. The non-breeding movements of 19 adult Cory’s shearwaters (*Calonectris borealis*) tracked by GLS from breeding colonies on Islas Canarias (28° 6′ N, 15° 24′ W) and the Azores (37° 44′ N, 25° 40′ W), and 9 Scopoli’s shearwater (*Calonectris diomedea*) tracked from Islas Baleares (39° 30′ N, 3° 0′ E) between 2000 and 2005 were extracted from the Birdlife International Global Procellariiform Tracking Database and from González-Solís *et al.* [5,6]. Birds were selected at random, away from the edge of the colony, and caught under appropriate regional licences. Loggers were attached to a plastic ring using cable ties and fitted to the tarsus, total device mass did not exceed 10g or 5% of body mass for any species.

Positions were calculated from logger data following standard methods [7]. Timings of sunset and sunrise were estimated according to set thresholds in the light curves recorded by the logger, and latitude derived from day length and longitude from the timing of local midday and midnight with respect to Greenwich Mean Time and Julian day. Data were filtered to remove locations derived from interrupted dawn or dusk events, or during the vernal and autumnal equinoxes; providing two positions per day with an accuracy of approximately 200 km [7]. Locations beyond 500 km from the breeding colonies [8,9]were classified as non-breeding in this analysis and provide the departure and arrival times in Supplementary Table 1., except for great skua locations taken from Magnusdottir *et al.* [2] which had been previously filtered to the known winter period of skuas. All spatial data were projected to a custom Lambert Azimuthal Equal Area projection centred on the CCLME (24° 38′ N, 15° 0′ W).

To estimate overlap with the CCLME we counted the number of days each individual spent in the CCLME and divided this by the number of days that individual was tracked for during the non-breeding period.

To identify multi-species foraging areas we then constructed spatial density maps by binning location data into a 200 km tessellated grid of hexagons covering the Atlantic. The size of the hexagons was chosen to be comparable to the error associated with geolocation logger data [7]. We chose species richness as an easily interpretable measure of biodiversity, and calculated it by summing the number of species that occurred in each hexagon. We only considered one winter period per individual.

Winter seasonal climatology composites (2002-2010) of sea surface temperature (SST, °C) and chlorophyll *a* (CHL, mg m^-3^) from the MODIS instrument onboard the Aqua (EOS PM) satellite (<http://oceancolor.gsfc.nasa.gov/>, Supplementary Figure 1) were only available for the period 21 December to 20 March. While this does not exactly match the temporal resolution of the location data it is the best composite available. We also included a measure of null usage as a parameter in the GAM that incorporated both habitat availability and sampling effort, as this was not uniform across species. The usage **u** of cell *x* under the null hypothesis was estimated (Equation 1) as a function of the biological distance *d* from cell *x* to colony *j*; where *σ ^2^* was the variance in step length for species *i*; and *n* the number of individuals of species *i* tracked from colony *j*.

*Equation 1*  $\boldsymbol{u}_{x}= \sum_{All i} \sum_{All j} \left( {n e}^{-\frac{{dx}_{j}^{2}}{{2\sigma}_{i}^{j}}} \right)$

**Supplementary Table 1.** Summary statistics for the tracking data by species including the departure and arrival dates to the breeding colonies. Values represent mean ± SD with the range in parenthesis.

| **Proportion in CCLME** | 0.09 ± 0.10  (0 - 0.25) | 0.26 ± 0.29  (0 - 0.75) | 0.12 ± 0.24  (0 - 0.68) | 0.10 ± 0.18  (0 - 0.77) | 0.35 ± 0.28  (0.06 - 0.74) | 0.08 ± 0.01  (0.06 - 0.09) | 0.04 ± 0.07  (0 - 0.31) | 0.24 ± 0.22  (0.01 - 0.68) | ^*^ |
| --- | --- | --- | --- | --- | --- | --- | --- | --- | --- |
| **Locations in CCLME** | 21.0 ± 24.0  (0 - 63) | 25.7 ± 29.8  (0 - 88) | 11.1 ± 21.8  (0 - 63) | 13.8 ± 23.2  (0 - 99) | 35.4 ± 36.9  (8 - 96) | 22.3 ± 3.1  (17 - 27) | 8.7 ± 14.3  (1 - 67) | 62.8 ± 51.8  (1 - 150) |  |
| **Number of locations** | 151.1 ± 20.8  (113 - 176) | 89.6 ± 15.4  (58 - 136) | 91.9 ± 0.4  (91 - 93) | 131.5 ± 27.4  (27 - 165) | 102.3 ± 39.6  (17 - 131) | 228.6 ± 18.6  (202 - 258) | 176.3 ± 21.6  (102 - 208) | 181.3 ± 64.0  (32 - 269) |  |
| **Winter period** | 208.4 ± 27.4  (182 - 248) | 93.6 ± 13.4  (66 - 135) | 92 | 133.9 ± 29.9  (26 - 192) | 104.5 ± 40.8  (16 - 131) | 287.9 ± 12.7  (263 - 300) | 237.2 ± 35.1  (161 - 340) | 254.3 ± 67.0  (140 - 357) |  |
| **Colony return date** | 8 Mar ± 24.4  (16 Feb – 8 Mar) | 25 Jan ± 11.2  (6 Jan - 25 Jan) | 31 Jan | 22 Mar ± 57.1  (28 Feb - 22 Mar) | 30 Apr ± 121.7  (10 Feb - 30 Apr) | 1 Jun ± 14.1  (3 May - 1 Jun) | 24 Nov ± 12.9  (28 Oct - 24 Nov) | 22 Apr ± 69.4  (15 Jan – 22 Apr) |  |
| **Colony departure date** | 12 Aug ± 8.0  (3 Aug - 27 Aug) | 23 Oct ± 7.4  (9 Oct - 17 Nov) | 1 Nov | 20 Oct ± 7.1  (14 Oct - 4 Nov) | 27 Oct ± 3.2  (22 Oct - 31 Oct) | 17 Aug ± 4.1  (13 Aug - 24 Aug) | 1 Apr ± 31.7  (5 Jan - 24 Jun) | 11 Jul ± 38.9  (20 May – 3 Sep) |  |
| **Percentage visiting CCLME** | 71.4 | 58.8 | 25.0 | 57.9 | 100.0 | 100.0 | 100.0 | 100.0 |  |
| **Species (n)** | Lesser black-backed gull  (7) | Northern gannet (34) | Great skua^*^  (16) | Cory’s shearwater  (19) | Scopoli’s shearwater  (9) | Sabine’s gull  (7) | South polar skua  (19) | Common tern  (12) |  |

**Supplementary Figure 1** Spatial patterns of habitat data; winter seasonal climatology composites (21 December to 20 March) of (a) sea surface temperature (SST, °C) and (b) chlorophyll *a* distribution (CHL, mg m^-3^) for the period 2002-2010.


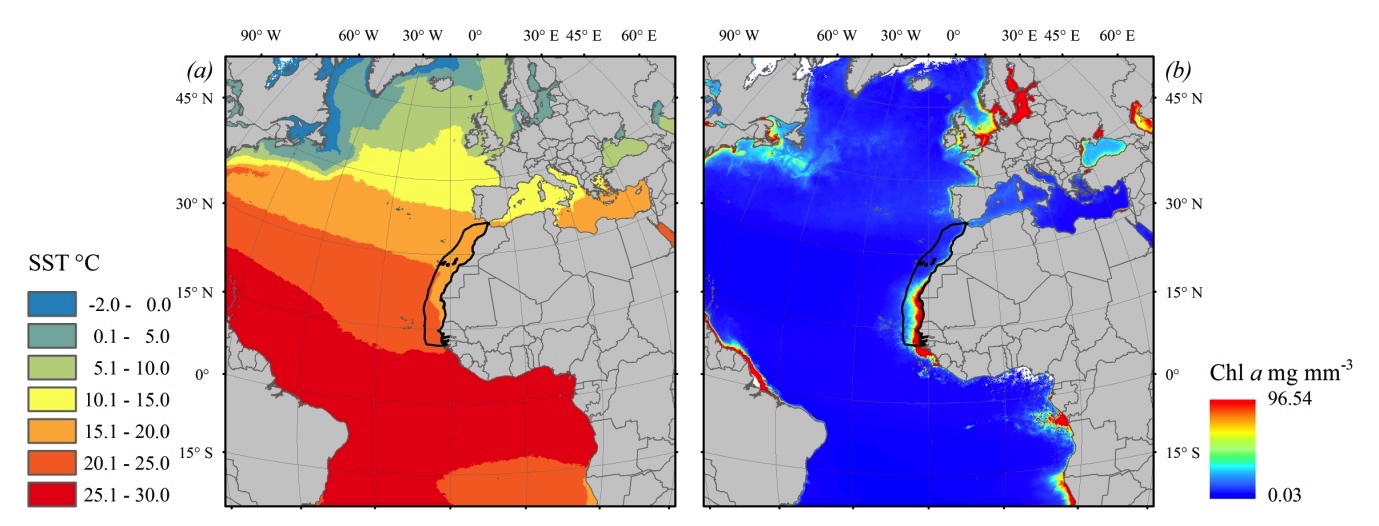


**Supplementary Figure 2** Non-breeding distributions of 8 seabird species tracked from colonies across the Atlantic between 2000 and 2011; *(a)* Cory’s shearwaters (*Calonectris borealis*); *(b)* Scopoli’s shearwater (*C. diomedea*); *(c)* lesser black-backed gulls (*Larus fuscus*); *(d)* northern gannets (*Morus bassanus*); *(e)* great skuas (*Stercorarius skua*); *(f)* south polar skuas (*S. maccormicki*); *(g)* common terns (*Sterna hirundo*); and *(h)* Sabine’s gulls (*Xema sabini*). Colours represent number of tracked individuals occurring in each hexagon; dark line represents the boundary of the Canary Current Large Marine Ecosystem; dots represent colonies of origin.


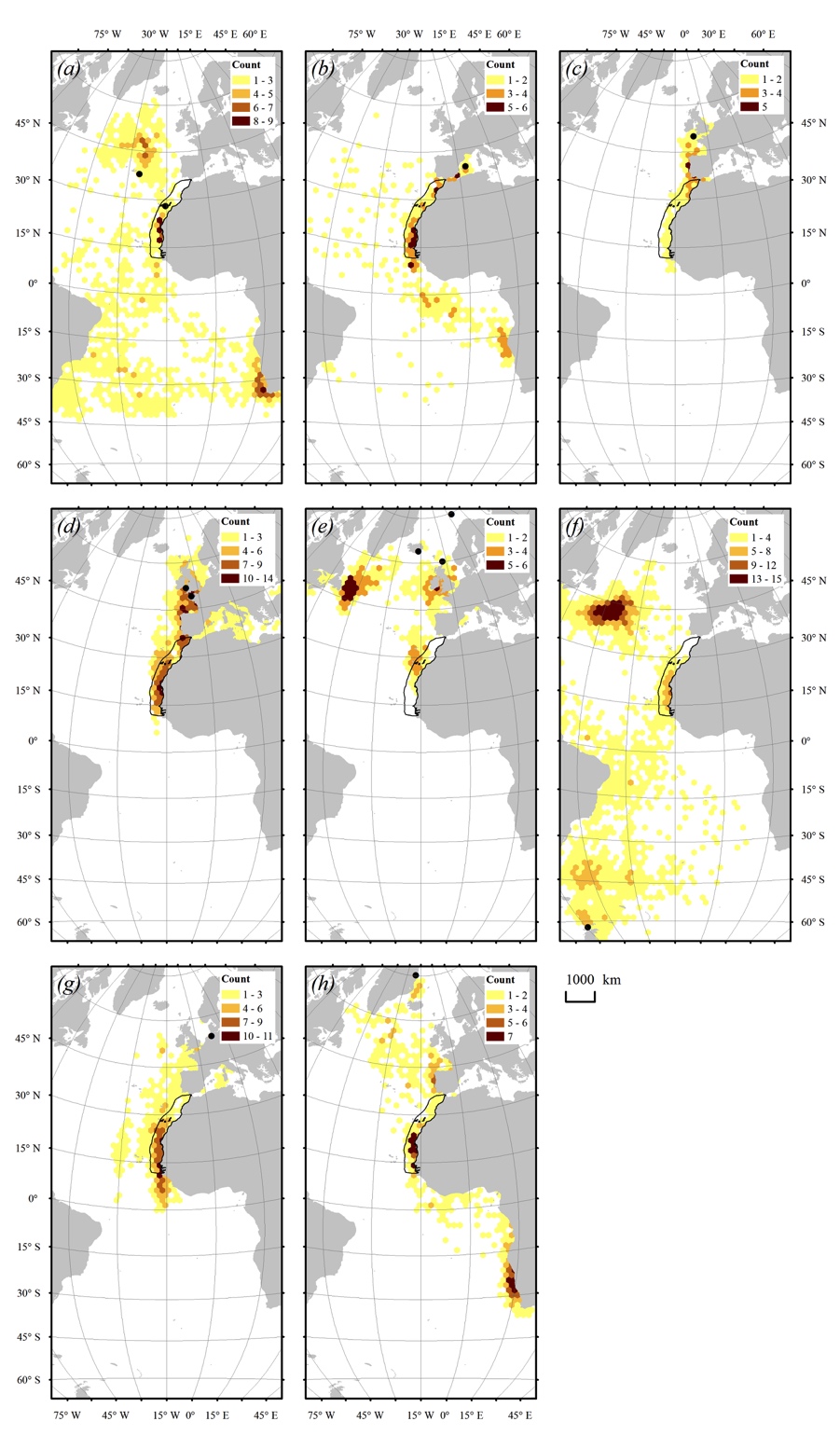


**References**

1. Stenhouse, I. J., Egevang, C. & Phillips, R. A. 2012 Trans-equatorial migration, staging sites and wintering area of Sabine’s gulls *Larus sabini* in the Atlantic Ocean. *Ibis (Lond. 1859).* **154**, 42–51.

2. Magnusdottir, E. et al. 2012 Wintering areas of Great Skuas *Stercorarius skua*  breeding in Scotland, Iceland and Norway. *Bird Study* , 37–41.

3. Kopp, M., Peter, H. U., Mustafa, O., Lisovski, S., Ritz, M. S., Phillips, R. A. & Hahn, S. 2011 South polar skuas from a single breeding population overwinter in different oceans though show similar migration patterns. *Mar. Ecol. Prog. Ser.* **435**, 263–267. (doi:10.3354/meps09229)

4. Becker, P. H., Schmaljohann, H., Riechert, J., Wagenknecht, G., Zajková, Z. & González-Solís, J. 2016 Common Terns on the East Atlantic Flyway: temporal–spatial distribution during the non-breeding period. *J. Ornithol.* (doi:10.1007/s10336-016-1346-2)

5. BirdLife 2010 The Global Procellariiform Tracking Database. **2011**.

6. González-Solís, J., Croxall, J. P., Oro, D. & Ruiz, X. 2007 Trans-equatorial migration and mixing in the wintering areas of a pelagic seabird. *Front. Ecol. Environ.* **5**, 297–301.

7. Phillips, R. A., Silk, J. R. D., Croxall, J. P., Afanasyev, V. & Briggs, D. R. 2004 Accuracy of geolocation estimates for flying seabirds. *Mar. Ecol. Ser.* **266**, 265–272.

8. Votier, S. C., Bearhop, S., Witt, M. J., Inger, R., Thompson, D. R. & Newton, J. 2010 Individual responses of seabirds to commercial fisheries revealed using GPS tracking, stable isotopes and vessel monitoring systems. *J. Appl. Ecol.* **47**, 487–497.

9. Guilford, T. C., Meade, J., Willis, J., Phillips, R. A., Boyle, D., Roberts, S., Collett, M., Freeman, R. & Perrins, C. M. 2009 Migration and stopover in a small pelagic seabird, the Manx shearwater Puffinus puffinus: insights from machine learning. *Proc. R. Soc. B-Biological Sci.* **276**, 1215–1223. (doi:10.1098/rspb.2008.1577)
